# Band-Engineered Local Cooling in Nanoscale Junctions

**DOI:** 10.1038/srep42647

**Published:** 2017-02-15

**Authors:** Bailey C. Hsu, Yu-Chang Chen

**Affiliations:** 1 Department of Electrophysics, National Chiao Tung University, 1001 University Road, Hsinchu 30010, Taiwan

## Abstract

The stability and performance of nanoscale junctions are closely related to the local effective temperature. The local effective temperature is mainly caused by the competition between heating and cooling processes in inelastic electron-phonon scat- tering. Local cooling occurs when the rate of energy in cooling exceeds that in heating. Previous research has been done using either specific potential configuration or an adatom to achieve local cooling. We propose an engineer-able local-cooling mechanism in asymmetric two-terminal tunneling junctions, in which one electrode is made of metal, whereas the other is made of a selectable bad-metal, such as heavily-doped polysilicon. The width of energy window of the selectable material, defined as the width covering all possible energy states counting from the conduction band minimum, can be engineered through doping. Interestingly, we have shown that substantial local cooling can be achieved at room temperature when the width of energy window of the low-density electrode is comparable to the energy of the phonon. The unusual local cooling is caused by the narrowed width of energy window, which obstructs the inelastic scattering for heating.

The phenomenon of local heating, which could occur when the current flows through electronic components, heats up devices and deteriorates the performance. As we continuously reach for the miniaturization of electronic devices, heat management has become undoubtedly a challenge for the tech industry^1^,^2^. The electron-phonon interaction is the major heating mechanism in junctions, whereas the electron-electron interaction has a minor influence; for example, the effect of the electron-electron interaction on the quantum point contact is nearly one order of magnitude smaller than the effect of the former^3^,^4^. Electrons traversing through the electronic components can lose (or gain) energy to excite (or relax) atom vibration in the device region. Typically, the net effect is heating up the device if the power of heating surpasses the power of cooling, and thereby causes local heating^5^,^6^,^7^,^8^,^9^,^10^,^11^,^12^. When the size is smaller than the mean-free-path of the electron-phonon interaction, the chance of inelastic scattering is reduced. Thus, heating is suppressed. This approach has resulted in the rapid development of nanoelectronics, which covers super-miniature electronic components formed by a nano-structure that connects the electrodes^13^,^14^,^15^,^16^,^17^,^18^,^19^. The characteristics of the electron-phonon interaction that causes heating in the bulk materials are distinguishable from those of the nanojunction devices. The acoustic phonons in the bulk crystal materials are goldstone modes. Phonons can be excited without the cost of minimum energy. By contrast, nanoscale junctions are characterized by inter-atomic interactions that break the translational symmetry. Electrons require a certain minimum amount of energy to overcome the gap and excite the phonon, which implies that local heating can be further reduced by operating a nanoscale device at a relatively low voltage, in which only a few thermally-excited high-energy electrons have sufficient energy to excite the phonons.

Although many extensive studies have investigated local heating in nanoscale junctions, very few research proposed potential local cooling mechanisms^20^,^21^,^22^,^23^,^24^,^25^,^26^. Some of these cooling mechanisms are Kramer Barrier crossing mechanism; an adatom attached to the atomic wire, which can enhance the rate of cooling under certain limits; and a metallic nanowire connected to superconductor leads coupling to longitudinal phonon modes. In this Letter, we propose a band-engineered two-terminal tunneling device model for local cooling, as shown in Fig. 1(a), in which the material for one side of the electrode is metal, e.g., gold, and the material for the other electrode is experimentally selectable. A nano-structure (e.g., an atom chain, a molecule, or a quantum dot, etc) bridges the asymmetric electrodes. We first define the term width of energy window as the width covering all possible energy states counting from the conduction band minimum. For simplicity, we considered a typical vibrational mode of the nano-structure [Fig. 1(b)], which has an energy *ħω* comparable to the width of the energy window of the bad metals, such as heavily-doped polysilicon which has a width of energy window controllable by doping concentration. We consider four major types of electron-phonon scattering processes [Fig. 1(c–f)]. Each type consists of its time-reversal processes as a pair. The net effect of heating (red lines) and cooling (blue lines) processes are responsible for the local heating. These phonon emission/absorption process diagrams describe the electrons travel from the right or left electrode gain (lose) energy to relax (cool down) or excite (heat up) phonons in the nano-structure. Heating power and cooling power are in a tug-of-war. In general, the pair of time reversal processes ensures that the local temperature is the same as the electrode environment temperature at zero bias. When an external bias is applied, a window between the Fermi levels of two electrodes is opened for net current. The current-induced power of heating typically surpasses the power of cooling, thus winning the competition and causing local heating. Nevertheless, we show when the width of energy window of the controllable materials is comparable to the energy of the phonon, the current-induced heating processes could be blocked when the electron loses energy and scatters to an energy lower than the band bottom. As such, the cooling power could surpass the heating power and could cause a condition such that the local effective temperature in the scattering region of the device is lower than the electrodes. Local cooling decreases the temperature of the electronic components by facilitating electron-vibration interactions, which are the same mechanisms that cause local heating and inelastic electron tunneling spectroscopy (IETS)^27^,^28^. By contrast, thermoelectric cooling (Peltier effect) employs the slope of the transmission function to cool the electrode region rather than device region^29^,^30^.

## Results

**Figure 2:**
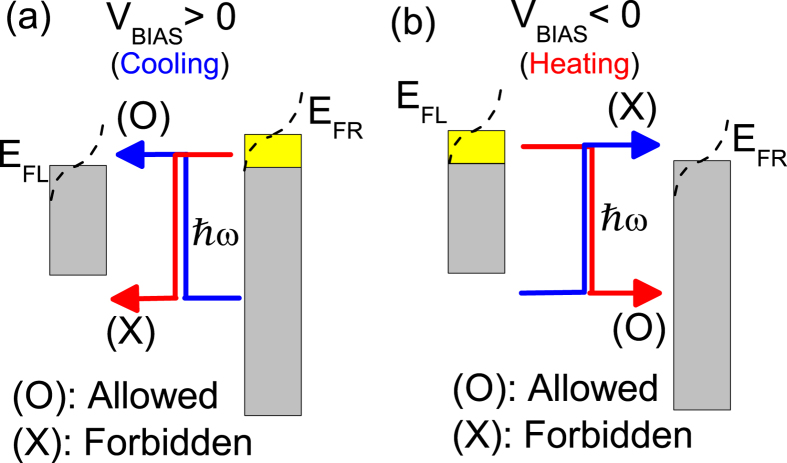
Mechanism of band-engineered local cooling and heating when an external voltage *V*_BIAS_ is applied. Here, we draw a representative major heating process and its associated cooling process opened by *V*_BIAS_. (**a**) For *V*_BIAS_ > 0, heating (cooling) process is forbidden (allowed) in favor of cooling. (**a**) For *V*_BIAS_ < 0, heating (cooling) process is allowed (forbidden) in favor of heating. The *ħω* refers to the energy of phonon. *E*_FL_/*E*_FR_ refer to the Fermi level of the left/right electrode. The dashed black line refers to the thermal broadening from the Fermi-Dirac distribution function.

In all the results presented here, the selected width of energy window of the left controllable material is much narrower than that of the right electrode made of gold. For example, one can choose bad metal as the controllable material, such as heavily-doped polysilicon. Polysilicon has a width of energy window determined by the carrier concentration which can be modulated by doping. The width of energy window can be expressed as 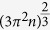, where *n* is the electron charge density controllable by doping related to the Wigner-Seitz radius 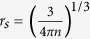. The mechanism of the band-engineered local cooling (heating) phenomenon is demonstrated in the band diagram of the tunneling junction, as shown in Fig. 2, where an energy window, 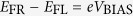, is open by the applied bias voltage. A pair of heating and cooling processes dominating the contribution of heating and cooling is also presented. When *V*_BIAS_ > 0, the heating process (red line) describes an electron scattered to an energy smaller than the bottom of the left band, as shown in Fig. 2(a). This heating process is prohibited by the narrow band because there are no available states below the band bottom, while their is no such obstruction for the corresponding cooling process. Consequently, heating is suppressed and the net power favors cooling. On the contrary, the cooling process is prohibited in favor of heating when *V*_BIAS_ < 0, as shown in Fig. 2(b), where the state for cooling process has an energy smaller than the left band bottom of the left electrodes. Consequently, cooling is enhanced for *V*_BIAS_ > 0, while heating is enhanced for *V*_BIAS_ < 0. This results in local cooling phenomenon, which occurs when *V*_BIAS_ > 0. To simplify the calculations, we assumed that the electron-vibration coupling is energy-independent, and we defined a scattering ratio 
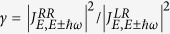
, namely the ratio between the backscattering and the forward scattering strength.

**Figure 3:**
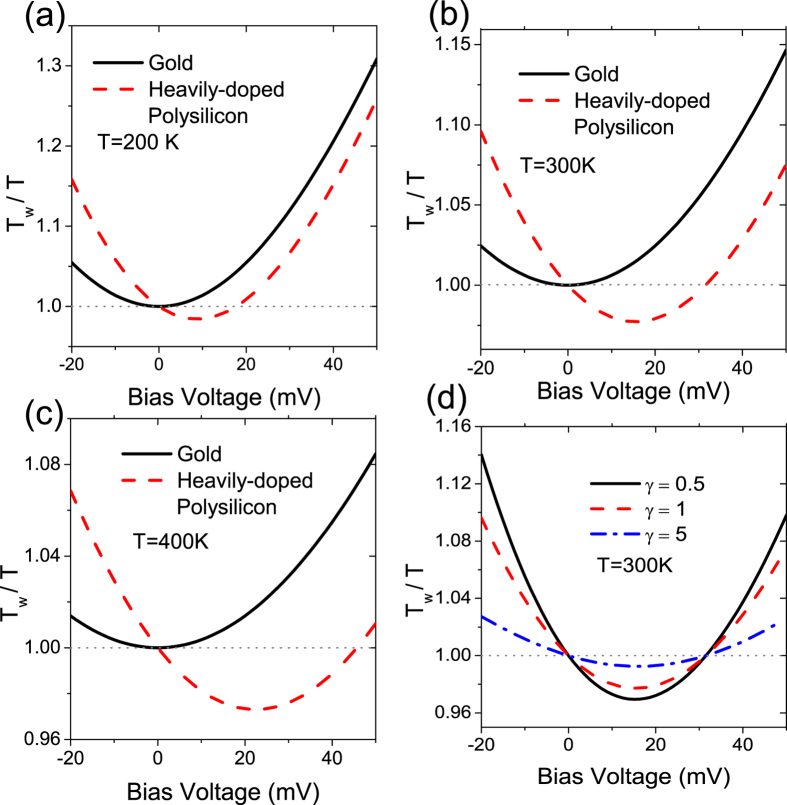
Temperature ratio (*T*_*w*_/*T*) vs. bias voltage with *γ* = 1 for the electrode temperature *T* = (**a**) 200 (**b**) 300, and (**c**) 400 K for the controllable materials: gold (black lines) and bad metal, e.g. heavily-doped polysilicon (red dashed lines) material. The dot (gray) line refers to the local temperature that is the same as the electrode temperature (*T*_*w*_/*T* = 1.0). (**d**) Temperature ratio (*T*_*w*_/*T*) vs. bias voltage with *γ* = 0.5, 1 and 5 at 300 K for heavily-doped polysilicon asymmetric electrodes.

In Fig. 3, we set the energy of phonon to *ħω* = 10 meV, a typical normal-mode energy for the gold point contact^9^. Figure 3(a) shows *T*_*w*_/*T* as a function of bias for the symmetric and asymmetric electrodes, where *T* = 200 K is the electrode (background) temperature. For the symmetric electrodes, the controllable material used is also gold (concentration *n* ≈ 5.97 × 10^22^ cm^−3^; width of energy window is around 5.566 eV). At zero bias, *T*_*w*_ = *T*. Local heating always occurs when bias is applied. We observe that *T*_*w*_/*T* (black line) is symmetric for the negative and positive biases in the symmetric junction. When the controllable material is heavily-doped polysilicon (here we set *n* ≈ 1 × 10^19^ cm^−3^ with the width of energy window around 1.69 × 10^−2^ eV), the symmetry is broken at all temperatures. Comparing with the symmetric electrodes, we observe that the local cooling is enhanced in positive bias regime, whereas local heating is enhanced in negative bias regime. For heavily-doped polysilicon electrode, the local temperature *T*_*w*_ (red dashed line) starts to decrease as *V*_BIAS_ increases until it reaches 9 mV. The local temperature is cooled and is lower than the background temperature at a *V*_BIAS_ roughly between 0 mV to 20 mV. Similar behavior can be extended to high electrode temperatures, as shown in Fig. 3(a–c), where *T* = 200, 300, and 400 K, respectively. As electrode temperature *T* increases, the minimum of the local temperature drops, and the range of cooling prolongs. In Fig. 3(d), a qualitative comparison of the strengths of the backscattering *γ* at *T* = 300 K with heavily-doped polysilicon electrode is plotted. The backscattering strength affects the overall heating and cooling rates, such that an increase in the bias voltage decreases the heating and cooling rates under large backscattering strength.

## Discussion

**Figure 4:**
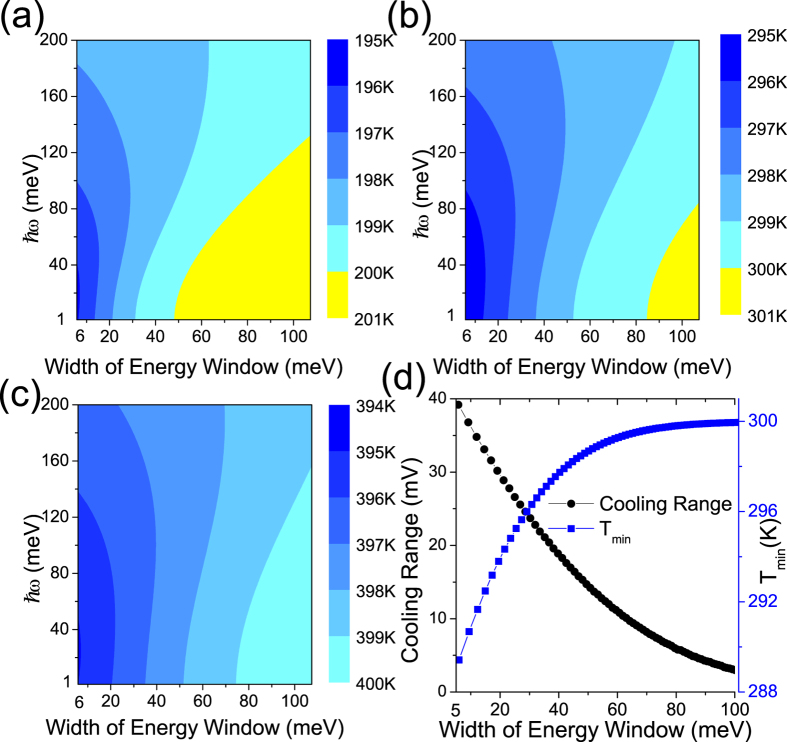
Contour plots of the local temperature with varying material widths of energy window and junction vibration mode energy *ħω*, and the electrode temperature set at (**a**) 200 (**b**) 300, and (**c**) 400 K. (**d**) The minimum local temperature (blue line with circle) and the range of local cooling (black line with square) scanned from 1 mV to 10 mV with different material widths of energy window at an electrode temperature of 300 K and scattering ratio *γ* = 1.

Given a certain vibration mode, what is the optimal material widths of energy window that can provide local cooling in the junction? To answer this question, we set the bias at 5 mV and created contour plots in which the local temperature is plotted as a function of the width of energy window and the normal-mode energy in Fig. 4(a–c), where the electrode temperature T is 200, 300, and 400 K, respectively. The blue color region represents a cooling range, where the local temperature of the device region is cooled to a temperature lower than the electrode (background) temperature. When the energy of vibration mode is fixed at 10 meV, we observe that the width of energy window required to attain local cooling increases when the electrode background temperature increases. When we compare the maximum width of energy window to have local cooling with the vibration mode energy set at *ħω* = 10 and 100 meV in Fig. 4(a–c), we observed that the corresponding widths of energy window can be much greater (for *ħω* = 10 meV) or smaller (for *ħω* = 100 meV) than the vibration mode energy *ħω*. This behavior may be owing to broadening by the rise of electrode temperature. In the case of *ħω* = 10 meV, the broadening is wide enough to allow heating processes from states below the Fermi-level to contribute, as in Fig. 2(a). Thus, heating will not be blocked for those states down below the Fermi-level. Therefore, the corresponding width of energy window is greater than the *ħω*. Similarly, in the case of *ħω* = 100 meV, the broadening of the Fermi-Dirac distribution is not wide enough to allow substantial heating processes to contribute from states below the Fermi-level. Therefore, the heating process can be blocked from the states above the Fermi-level in Fig. 2(a), resulting in a width of energy window smaller than the *ħω*. Next, we examined the optimization condition: what are the lowest temperature and the cooling range for specific experimentally controllable settings. We perform a search of the cooling range and the minimum local temperature as a function of the material width of energy window, as shown in Fig. 4(d), where we set *T* = 300 K and *γ* = 1. From the plot, we observed that the minimum local temperature decreased, and the cooling range is increased when the material width of energy window was reduced.

In summary, we presented a band-engineered approach to reach local cooling in asymmetric tunneling junctions. By selecting a pair of high-density and low-density metals as electrodes, substantial local cooling effect can be achieved. In our proposed setup, the charge density of low density material could be modulated by doping. We observe that positive bias favors cooling and negative bias favors heating. We also demonstrate that the range of cooling and its lowest local temperature attained can be controlled and optimized. This provides significant insights for future smart device designs, with regard to being self-cooled to improve stability, energy consumption, and performance. Major advantages of this band engineered approach are its controllability, wide applicability, and easy implementation in experiments without the need to match a specific potential with certain nano-structure configurations.

## Methods

We start with a Hamiltonian *H* that describes the inelastic electron-vibration interaction,


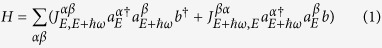


where *α, β* = {*L, R*}; *b* is the phonon annihilation operator, and 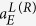 is the annihilation operator for electrons; 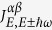 is the coupling strength between the electrons and the vibration of the junction atom. Two terms in the summation describe a pair of heating and cooling processes. Note that 
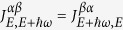
 because of the time-reversal symmetry. Also we assume the inelastic electron-vibration interactions follow Markovian process. The rate of energy absorbed (emitted) by the anchored nano-structures due to the incident electrons from *β* = {*L, R*} electrode and scattered to the *α* = {*L, R*} electrode via the vibrational mode excitation/relaxation can be calculated from Fermi golden rule and is denoted by 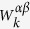, which has the form of


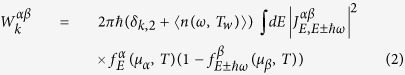


where 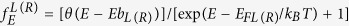 is the Fermi-Dirac distribution function describing the statistic of electrons in the left (right) electron reservoir with temperature *T*, Fermi energies *E*_*FL(R*)_, and the bottom of the conduction band *Eb*_*L(R*)_; The Heaviside function, *θ(E* − *Eb*_*L(R*)_), describes no available state below the conduction band; 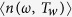 is the average number of local phonons and can be expressed as 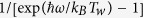 where *T*_*w*_ refers to the effective local (wire) temperature and *k*_*B*_ is the Boltzmann’s constant; *δ*_*k*,2_ is the Kronecker delta, where *k* = 1(2) corresponds to the relaxation (excitation) of the vibrational mode. The range of integration in Eq. (2) includes all energies within the energy band of the electrodes. Note that a process of an electron scattered to an energy below the bottom of the conduction band is not allowed. Contributions of these prohibited scattering events must be excluded from the energy integration. Temperature is a well-defined quantity for a system in equilibrium state. It can be extended to a non-equilibrium system in a steady state. When a steady state current is reached, the local temperature *T*_*w*_ is well-defined: *T*_*w*_ can be solved by the net power *P* = 0, where 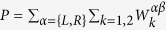. Please see supplemental information for more detailed theoretical formalism.

## Additional Information

**How to cite this article**: Hsu, B. C. and Chen, Y.-C. Band-Engineered Local Cooling in Nanoscale Junctions. *Sci. Rep.* **7**, 42647; doi: 10.1038/srep42647 (2017).

**Publisher's note:** Springer Nature remains neutral with regard to jurisdictional claims in published maps and institutional affiliations.

## Supplementary Material

Supplementary Information

## Figures and Tables

**Figure 1:**
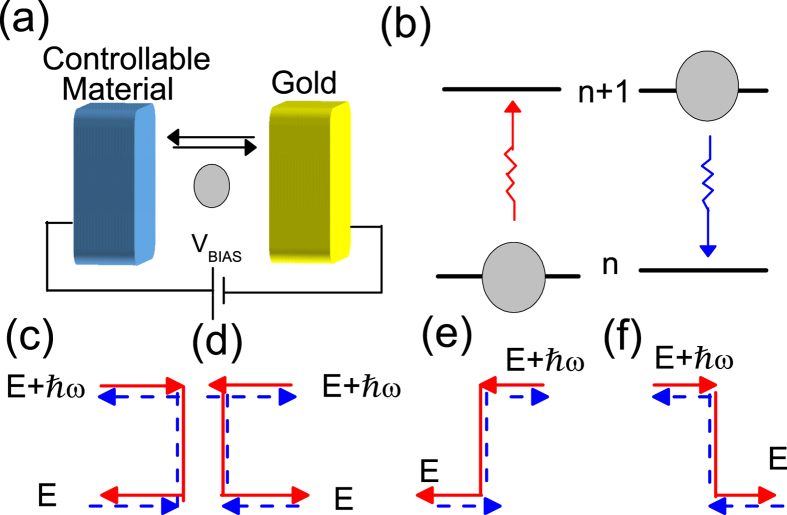
(**a**) Schematic of the proposed asymmetric tunneling junction with a nano-structure bridging electrodes connected to an external bias. The material and the width of energy window of the left electrode are experimentally controllable. (**b**) Phonon mode of the nano-structure is excited (heated) and relaxed (cooled) by the four pairs of time-reversal inelastic scattering processes depicted in (**c**–**f**). Each pair describes the electron incident from the left/right electrode, and subsequently inelastically scattered to the left/right electrode, where red/blue (solid/dashed) line refers to the pair of heating/cooling processes.
