# Supplementary material for: Band-Engineered Local Cooling in Nanoscale Junctions
Source: Sci Rep. 2017 Feb 15;7:42647. doi: 10.1038/srep42647 (PMC5309743; doi:10.1038/srep42647)
Supplement: Supplementary Information [file srep42647-s1.pdf]

Supplementary Information for Band-Engineered Local Cooling in Nanoscale Junctions

Bailey C. Hsu[1] and Yu-Chang Chen[1,*]

[1] Department of Electrophysics, National Chiao Tung University, Hsinchu, TAIWAN

*Corresponding Authors: yuchangchen@mail.nctu.edu.tw

Supplementary information (SI) shows the details in local-cooling theory.

A. Hamiltonian of Electron–Vibration Interactions:

We study the electron–vibration interaction in second quantization form. To consider the second quantization Hamiltonian for the electron–vibration interaction in an non–equilibrium nanoscale junction, we consider a field operator that is constructed from the current–carrying effective single–particle wave function obtained in the framework of density functional theory in scattering approaches,

$$\hat{\Psi} = \sum_{\alpha,E,\mathbf{K}} a^{\alpha}_{E\mathbf{K}}(t)\Psi^{\alpha}_{E\mathbf{K}}(\mathbf{r}), \tag{1}$$

where $\alpha = L$ and R; effective single–particle wavefunctions $\Psi^{L(R)}_{E\mathbf{K}}(\mathbf{r})$ describes electrons incident from the left and right electrodes; $a^{L(R)}_{E\mathbf{K}}(t) = \exp(-i\omega t)a^{L(R)}_{E\mathbf{K}}$; and $a^{L(R)}_{E\mathbf{K}}$ is the annihilation operators of electrons incident from the left (right) reservoir, satisfying the anti–commutation relations,

$$\{a^{\alpha}_{E_1\mathbf{K}_1}, a^{\beta\dagger}_{E_2\mathbf{K}_2}\} = \delta_{\alpha\beta}\delta(E_1 - E_2)\delta(\mathbf{K}_1 - \mathbf{K}_2), \tag{2}$$

where $\beta = L$ or R.

The expectation value of the product of electron creation and annihilation operator at thermal equilibrium is given by,

$$< a^{\alpha\dagger}_{E_1\mathbf{K}_1} a^{\beta}_{E_2\mathbf{K}_2} >= \delta_{\alpha\beta}\delta(E_1 - E_2)\delta(\mathbf{K}_1 - \mathbf{K}_2) f^{\alpha}_{E}, \tag{3}$$

where the statistics of electrons coming from the left (right) electrodes are determined by the equilibrium Fermi–Dirac distribution function $f^{L(R)}_{E} = 1/\{1 + \exp[(E - \mu_{L(R)})/(k_B T)]\}$ in the left (right) reservoir. The Lippmann–Schwinger equation allows the wave functions of the entire system to satisfy the same continuum normalization condition,

$$\int d\mathbf{r}[\Psi^{R(L)}_{E_1\mathbf{K}_1}(\mathbf{r})]^{*}\nabla\Psi^{R(L)}_{E_2\mathbf{K}_2}(\mathbf{r}) = \delta(E_1 - E_2)\delta(\mathbf{K}_1 - \mathbf{K}_2). \tag{4}$$

To consider the electron–vibration interactions, we start with a more general Hamiltonian including ions and the interaction between electrons and ions,

$$H = H_{el} + H_{ion} + H_{el-ion}. \tag{5}$$

where H_{el} is the electronic part of the Hamiltonian,

$$H_{el} = \sum_i \frac{\mathbf{p}_i^2}{2m_e} + \sum_{i<j} \frac{e^2}{|\mathbf{r}_i - \mathbf{r}_j|}, \tag{6}$$

where m_e is the electron mass, $\mathbf{p}_i$ is the momentum of the i–th electron, and $\mathbf{r}_i$ is the position of the i–th electron. H_{ion} is the ionic part of Hamiltonian,

$$H_{ion} = \sum_i \frac{\mathbf{P}_i^2}{2M_i} + \sum_{i<j} V_{ion}(\mathbf{R}_i - \mathbf{R}_j), \tag{7}$$

where M_i is the mass of the i–th ion, $\mathbf{P}_i$ is the momentum of i–th ion, $\mathbf{R}_i$ is the position of the i–th ion, and $V_{ion}(\mathbf{R}_i - \mathbf{R}_j)$ is the interaction between the i–th and j–th ions. H_{el-ion} describes the interaction between the i–th electron and j–th ions,

$$H_{el-ion} = \sum_{i,j} V_{ei}(\mathbf{r}_i - \mathbf{R}_j). \tag{8}$$

To consider the vibronic coupling, we start by considering small ionic oscillations,

$$\mathbf{R}_i = \mathbf{R}_i^0 + \mathbf{Q}_i, \tag{9}$$

where $\mathbf{Q}_i$ is a small deviation of position away from the equilibrium position for the i–th ion represented in the Cartesian coordinate system. Therefore,

$$V_{ion}(\mathbf{R}_i - \mathbf{R}_j) = V_{ion}(\mathbf{R}_i^0 - \mathbf{R}_j^0 - (\mathbf{Q}_i - \mathbf{Q}_j))$$
$$\approx V_{ion}(\mathbf{R}_i^0 - \mathbf{R}_j^0) + H_{osc}, \tag{10}$$

where the oscillatory part of ions H_{osc} is

$$H_{osc} = \frac{1}{2}\sum_{i<j} \sum_{\mu,\nu=x,y,z} \mathbf{F}_{\mu\nu} (\mathbf{Q}_i - \mathbf{Q}_j)_\mu (\mathbf{Q}_i - \mathbf{Q}_j)_\nu \mathbf{F}_{\mu\nu}$$
$$= \frac{\partial^2}{\partial \mathbf{R}_\mu \partial \mathbf{R}_\nu} V_{ion}(\mathbf{R}_i - \mathbf{R}_j)|_{\mathbf{R}_i=\mathbf{R}_i^0;\mathbf{R}_j=\mathbf{R}_j^0}. \tag{11}$$

Ion oscillations can be mapped into a set of independent simple harmonic oscillators via normal coordinates $\{q_j\}$, i.e., $(Q_i)_\mu = \sum_j A_{i\mu,j} q_j$. The oscillatory part of ions H_{osc} is diagonalized and has the form of

$$H_{osc} = \sum_i \frac{1}{2} \dot{q}_i \dot{q}_i + \sum_i \frac{1}{2} \omega_i q_i q_i, \tag{12}$$

where ω_i is the frequency of the i–th normal mode. One can introduce a canonical transformation, $\tilde{Q}_i = \sqrt{\frac{\omega_i}{2\hbar}} q_i$ and $\tilde{P}_i = \sqrt{\frac{\omega_i}{2\hbar}} \dot{q}_i$, which transforms H_{osc} to

$$H_{osc} = \frac{1}{2}\sum_i \hbar\omega_i (\tilde{P}_i^2 + \tilde{Q}_i^2), \tag{13}$$

where one has $[\tilde{Q}_j, \tilde{P}_k] = \frac{i}{2}\delta_{jk}$. Using $b_i = \tilde{Q}_i + i\tilde{P}_i$ and $b_i^\dagger = \tilde{Q}_i - i\tilde{P}_i$, the oscillatory part Hamiltonian can be second quantized by phonon creation and annihilation operators and become a set of independent simple harmonic oscillators,

$$H_{osc} = \sum_j \hbar\omega_j (b_j^\dagger b_j + \tfrac{1}{2}). \tag{14}$$

Next, we expand the electron–vibration interactions in terms of lowest order in small oscillation, $H_{el-ion} = H_{el-ion}^{eq} + \delta H_{el-ion}$, where we consider the interaction between electron and ion at nonlocal pseudopotential level,

$$\Delta H_{el-ion} = \sum_{i,\mu} (\mathbf{Q}_i)_\mu \cdot \frac{\partial}{\partial \mu} V^{ps}(\mathbf{r}, \mathbf{R}_i^0), \tag{15}$$

where $\frac{\partial}{\partial \mu} = \frac{\partial}{\partial \mathbf{R}^0_\mu}$ is the derivative with respect to the position of the i–th ion in $\mu = x, y,$and z directions. By using $(Q_i)_\mu = \sum_j A_{i\mu,j} q_j = \sum_j A_{i\mu,j} \sqrt{\frac{\hbar}{2\omega_j}} \left(b_j + b_j^\dagger\right)$ and placing M_i back into ΔH_{el-ion} , we obtain

$$\Delta H_{el-ion} = \sum_j \sum_i \sum_\mu \sqrt{\frac{\hbar}{2M_i\omega_j}} A_{i\mu,j}(b_j + b_j^\dagger) \frac{\partial}{\partial \mu} V^{ps}(\mathbf{r}, \mathbf{R}_i^0), \tag{16}$$

where we place back the mass of the i–th ions M_i. The orthonormal conditions for the canonical transformation between normal coordinates and Cartesian coordinates: $\sum_{i,\mu} A_{i\mu,j'} A_{i\mu,j'} = \delta_{j,j'}$. The vibronic coupling can be second quantized by applying the field operator

$$H_{el-vib} = \int \hat{\Psi}^\dagger(\mathbf{r})(\Delta H_{el-ion})\hat{\Psi}^\dagger(\mathbf{r}) d\mathbf{r}. \tag{17}$$

Finally,the many–body Hamiltonian of the system under consideration becomes $H = H_{el} + H_{vib} + H_{el-vib}$, H_{el} is the electronic part of the Hamiltonian under adiabatic approximations, and H_{vib} is the ionic part of the Hamiltonian, which can be casted into a set of independent simple harmonic oscillators via canonical transformation. H_{el-vib} is the part of the Hamiltonian for electron–vibration interactions that has the following form:

$$H_{el-vib} = \sum_{\alpha,\beta,E_1,E_2,j} \left(\sum_{i,\mu} \sqrt{\frac{\hbar}{2M_i\,\omega_j}} A_{i\mu,j} J^{i\mu,\alpha\beta}_{E_1\mathbf{K}_1,E_2\mathbf{K}_2} \right) a^{\alpha\dagger}_{E_1} a^{\beta}_{E_2} \left(b_j + b_j^\dagger\right). \tag{18}$$

The coupling constant $J^{i\mu,\alpha\beta}_{E_1,E_2}$ between electrons and the vibration of the i–th atom in μ $(= x, y, z)$ component can be calculated as

$$J^{i\mu,\alpha\beta}_{E_1,E_2} = \int d\mathbf{r} \int d\mathbf{r}' \int d\mathbf{K} [\Psi^{\alpha}_{E_1\mathbf{K}_1}(\mathbf{r})]^* [\partial_\mu V^{ps}(\mathbf{r}, \mathbf{r}', \mathbf{R}_i) \Psi^{\beta}_{E_2\mathbf{K}_2}(\mathbf{r}')], \tag{19}$$

where $V^{ps}(\mathbf{r}, \mathbf{r}', \mathbf{R}_i)$ is the nonlocal pseudopotential, which represents the interaction between electrons and the i–th ion.

The normal modes of the single Au atom connected to electrodes (ω_j) and the canonical transformation matrix $(A_{i\mu,j})$ are calculated with Gaussian09 code by freezing the positions of atoms located in the electrode region. Three normal modes (two degenerate transverse modes with energies $\hbar\omega_1 = \hbar\omega_2 = 4$ meV; one longitudinal mode with energy $\hbar\omega_3 = 10$ meV) are associated to the degrees of freedom of the single atom, as shown in Figure S1.

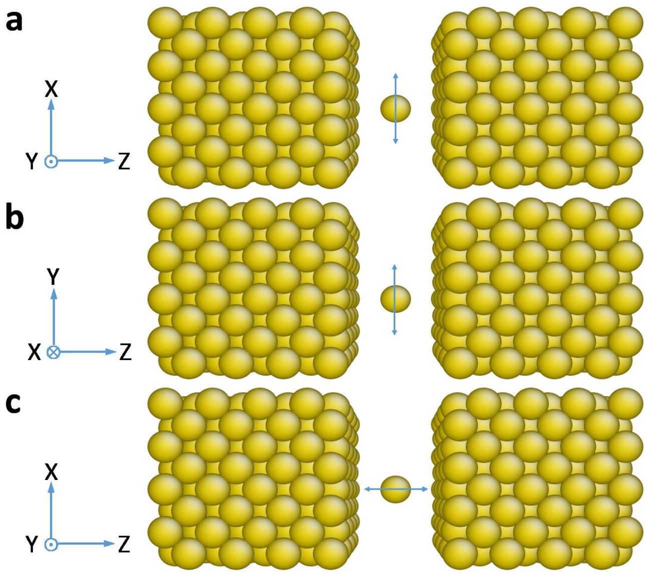

Figure S1: | **Normal modes of the gold point contact.**

a and **b** are two degenerate transverse normal modes with normal mode energies $\hbar\omega_1 = \hbar\omega_2 \approx 4$ meV. **c** is the longitudinal normal mode with energy $\hbar\omega_3 \approx 10$ meV

B. Theory of Local Heating and Cooling:

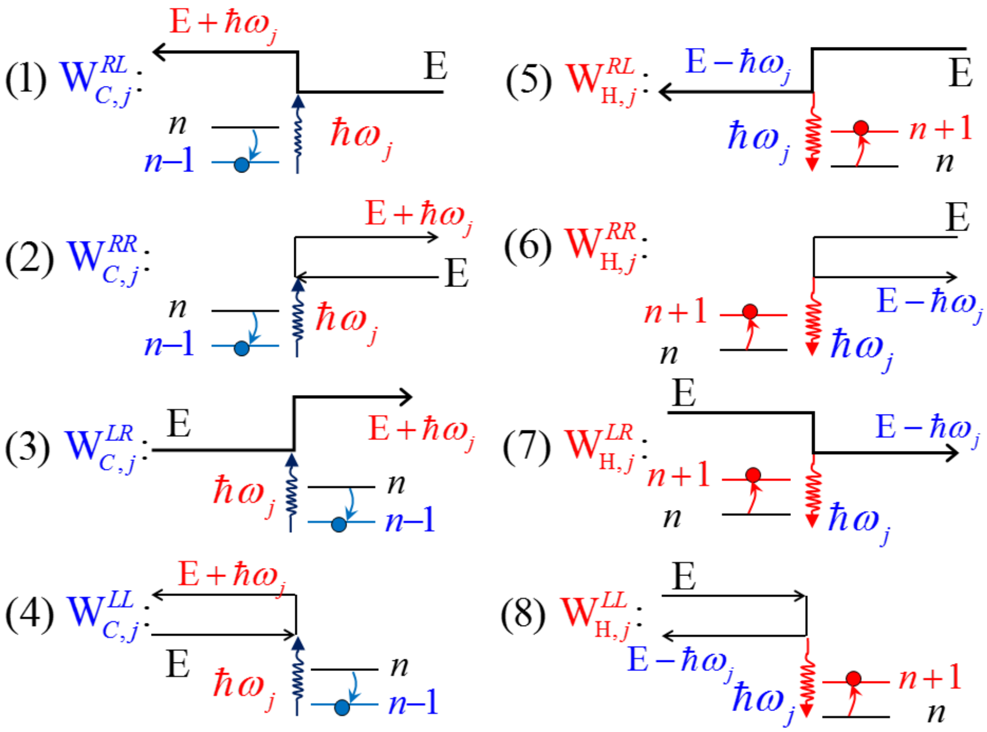

Figure S2: | **Schematic of a typical atomistic junction.**

Feynman diagrams of the first–order electron–vibration scattering mechanisms. Diagrams (1) to (4) shows the cooling processes, and diagrams (5) to (8) show the heating processes.

The rate of energy absorbed (emitted) by the anchored nano–structures due to incident electrons from the $\beta = \{L, R\}$ electrode and scattered to the $\alpha = \{L, R\}$ electrode via a vibrational mode j is denoted by $W_{H(C),j}^{\alpha\beta}$. The total thermal power generated in the junction P is the sum of contribution from eight first–order scattering processes (four heating and four cooling processes) and from all the vibrational modes shown in Figure **S2**,

$$P = \sum_{j \in vib} \left[\sum_{\alpha=\{L,R\}} \sum_{\beta=\{L,R\}} (W_{H,j}^{\alpha\beta} - W_{C,j}^{\alpha\beta})\right], \quad (20)$$

where the power for each process is estimated using the Fermi golden rule for all modes:

$$W_{H(C),j}^{\alpha\beta} = \sum_{j=\text{modes}} \sum_{E_i,E_f} \frac{2\pi}{\hbar} |\langle f|H_{el-vib}|i\rangle|^2 \left(E_f - E_i \pm \hbar\omega_j\right), \quad (21)$$

where the statistic average of state $|i\rangle = \left|\{\cdots, f_E^R, \cdots\}; \{\cdots, f_E^L, \cdots\}; \{\cdots, n(\omega_j, T_w), \cdots\}\right\rangle$ includes electron states of the left and right electrodes and local phonon states in the scattering region. Considering the eight scattering processes, one obtains

$$W_{H(C),j}^{\alpha\beta}(T_e, T_w, V_B) = \sum_{j=\text{modes}} 2\pi\hbar\left[\delta + n\left(\omega_j, T_w\right)\right]$$

$$\cdot \int dE (1 - f_{E\pm\hbar\omega_j}^{\alpha}) f_E^{\beta} \left|\sum_{i,\mu,j} A_{i\mu,j} J_{E\pm\hbar\omega_j,E}^{i\mu,\alpha\beta}\right|^2, \quad (22)$$

where $\delta = 1$ for heating processes and $\delta = 0$ for cooling processes which excite (relax) the normal mode vibration. $E + \hbar\omega_j$ is for cooling and $E - \hbar\omega_j$ is for heating processes.

When the system comes to thermal equilibrium, the rate of thermal energy, which is generated by the heating processes, balances the energy rate that is absorbed by the local processes. Thus, the temperature in the center scattering (T_w) can be obtained by solving $P = 0$. The current can be calculated from the wave function and can be further reduced to Landauer's formula

$$\mathrm{I} = \frac{2e}{h} \int dE (f_E^R - f_E^L) \tau(E) = \frac{2e^2}{h} \tau(E)\, \Delta V \approx \frac{2e^2}{h} \tau(E)\, S \Delta T$$

(23)

where$\tau(E)$ is the transmission function, S is the Seebeck coefficient, and ΔT is the induced temperature difference. Similarly, the heat current can be obtained via

$$\mathrm{J_{el}} = \frac{2}{h} \int dE \left(E - E_f\right)(f_E^R - f_E^L)\tau(E) \approx \kappa_{\mathrm{el}} \Delta T, \quad (24)$$

where κ_{el} the electric thermal conductance. Following from our previous work in Ref [1], after expanding the Seebeck coefficient and electric thermal conductance in the lowest order in the temperature, we can obtain

$$\mathrm{S} \approx \alpha\, \mathrm{T} \quad (25)$$

$$\kappa_{\mathrm{el}} \approx \beta\, \mathrm{T} \quad (26)$$

where $\alpha = -\frac{\pi^2 \kappa_B^2 \left(\frac{\partial \tau(\mu)}{\partial E}\right)}{3e^2}$ and $\beta = \frac{2\pi^2 \kappa_B^2 (\tau(\mu))}{3h}$.

By substituting Eq.(26) back to Eq.(24), the heat current can be expressed as,

$$\mathrm{J_{el}} \approx \frac{2\pi^2 \kappa_B^2 \tau(E)}{3h}\, T\, \Delta T. \quad (27)$$

Now we can find the ratio between the electric current and the heat current, namely J_{el}/I

$$\frac{J_{el}}{I} = \frac{\frac{\pi^2 \kappa_B^2}{3e^2} T}{S} = \frac{LT}{S} \approx \frac{L}{\alpha} \tag{28}$$

where L is the Lorentz number $\frac{\pi^2 \kappa_B^2}{3e^2}$.

[1] Ilias Amanatidis, Jing-Yao Kao, Li-Yang Du, Chun-Wei Pao, and Yu-Chang Chen, *J. Phys. Chem. C*, 2015, 119 (52), pp 28728–28736
